# Supplementary material for: A Simulation Framework for Modeling the Within-Patient Evolutionary Dynamics of SARS-CoV-2
Source: Genome Biol Evol. 2023 Nov 10;15(11):evad204. doi: 10.1093/gbe/evad204 (PMC10664409; doi:10.1093/gbe/evad204)
Supplement: evad204_Supplementary_Data [file evad204_supplementary_data.pdf]

Supplemental Table 1. List of parameter levels for all plausible models.

μ	Bottleneck Size	K	Infection Duration	Recombination	Ξ	Burst Size	DFE
Low	Low	Low	Lowest	n/a	n/a	n/a	n/a
Low	Low	Low	Lowest	Low	n/a	n/a	n/a
Low	Low	Low	Lowest	Low	Low	Low	n/a
Low	Low	Low	Lowest	Low	Low	Mid	n/a
Low	Low	Low	Lowest	Low	Mid	Low	n/a
Low	Low	Low	Lowest	Low	Low	Low	4:1
Low	Low	Low	Lowest	Low	Low	Mid	4:1
Low	Low	Low	Lowest	Low	Low	High	4:1
Low	Low	Low	Lowest	Low	Mid	Low	4:1
Low	Low	Low	Lowest	Low	Mid	High	4:1
Low	Low	Low	Lowest	Low	Low	Low	1:1
Low	Low	Low	Lowest	Low	Low	Mid	1:1
Low	Low	Low	Lowest	Low	Low	High	1:1
Low	Low	Low	Lowest	Low	Mid	Low	1:1
Low	Low	Low	Lowest	Low	Mid	Mid	1:1
Low	Low	Low	Lowest	Low	Mid	High	1:1
Low	Low	Low	Lowest	Low	Low	Low	1:4
Low	Low	Low	Lowest	Low	Low	Mid	1:4
Low	Low	Low	Lowest	Low	Low	High	1:4
Low	Low	Low	Lowest	Low	Mid	Mid	1:4
Low	Low	Low	Lowest	Low	Mid	High	1:4
Low	Low	Low	Lowest	Low	High	Mid	1:4
Low	Low	Low	Lowest	Low	High	High	1:4
Low	Low	Low	Lowest	Mid	n/a	n/a	n/a
Low	Low	Low	Lowest	Mid	Low	Low	n/a
Low	Low	Low	Lowest	Mid	Mid	Low	n/a
Low	Low	Low	Lowest	Mid	Low	Low	4:1
Low	Low	Low	Lowest	Mid	Mid	Low	4:1
Low	Low	Low	Lowest	Mid	Mid	High	4:1
Low	Low	Low	Lowest	Mid	Low	Low	1:1
Low	Low	Low	Lowest	Mid	Low	Mid	1:1
Low	Low	Low	Lowest	Mid	Low	High	1:1
Low	Low	Low	Lowest	Mid	Mid	Low	1:1
Low	Low	Low	Lowest	Mid	Mid	High	1:1
Low	Low	Low	Lowest	Mid	Low	Low	1:4
Low	Low	Low	Lowest	Mid	Low	Mid	1:4
Low	Low	Low	Lowest	Mid	Low	High	1:4
Low	Low	Low	Lowest	Mid	Mid	Low	1:4
Low	Low	Low	Lowest	Mid	Mid	Mid	1:4

μ	Bottleneck Size	K	Infection Duration	Recombination	Ξ	Burst Size	DFE
Low	Low	Low	Lowest	Mid	Mid	High	1:4
Low	Low	Low	Lowest	Mid	High	Low	1:4
Low	Low	Low	Lowest	Mid	High	Mid	1:4
Low	Low	Low	Lowest	Mid	High	High	1:4
Low	Low	Low	Lowest	High	Low	Low	n/a
Low	Low	Low	Lowest	High	Low	Low	4:1
Low	Low	Low	Lowest	High	Low	High	4:1
Low	Low	Low	Lowest	High	Mid	Low	4:1
Low	Low	Low	Lowest	High	Mid	High	4:1
Low	Low	Low	Lowest	High	Low	Low	1:1
Low	Low	Low	Lowest	High	Low	Mid	1:1
Low	Low	Low	Lowest	High	Low	High	1:1
Low	Low	Low	Lowest	High	Mid	Low	1:1
Low	Low	Low	Lowest	High	Mid	Mid	1:1
Low	Low	Low	Lowest	High	Mid	High	1:1
Low	Low	Low	Lowest	High	High	Low	1:1
Low	Low	Low	Lowest	High	Low	Low	1:4
Low	Low	Low	Lowest	High	Low	Mid	1:4
Low	Low	Low	Lowest	High	Low	High	1:4
Low	Low	Low	Lowest	High	Mid	Low	1:4
Low	Low	Low	Lowest	High	Mid	Mid	1:4
Low	Low	Low	Lowest	High	Mid	High	1:4
Low	Low	Low	Lowest	High	High	Low	1:4
Low	Low	Low	Lowest	High	High	Mid	1:4
Low	Low	Low	Lowest	High	High	High	1:4
Low	Low	Mid	Lowest	n/a	n/a	n/a	n/a
Low	Low	Mid	Lowest	Low	n/a	n/a	n/a
Low	Low	Mid	Lowest	Low	Low	Mid	n/a
Low	Low	Mid	Lowest	Low	Mid	Mid	n/a
Low	Low	Mid	Lowest	Low	High	Low	n/a
Low	Low	Mid	Lowest	Low	Low	Mid	4:1
Low	Low	Mid	Lowest	Low	Low	High	4:1
Low	Low	Mid	Lowest	Low	Mid	Low	4:1
Low	Low	Mid	Lowest	Low	Mid	Mid	4:1
Low	Low	Mid	Lowest	Low	High	Low	4:1
Low	Low	Mid	Lowest	Low	High	Mid	4:1
Low	Low	Mid	Lowest	Low	High	High	4:1
Low	Low	Mid	Lowest	Low	Low	Low	1:1
Low	Low	Mid	Lowest	Low	Low	Mid	1:1
Low	Low	Mid	Lowest	Low	Mid	Low	1:1
Low	Low	Mid	Lowest	Low	Mid	Mid	1:1

μ	Bottleneck Size	K	Infection Duration	Recombination	Ξ	Burst Size	DFE
Low	Low	Mid	Lowest	Low	Mid	High	1:1
Low	Low	Mid	Lowest	Low	High	Low	1:1
Low	Low	Mid	Lowest	Low	High	Mid	1:1
Low	Low	Mid	Lowest	Low	High	High	1:1
Low	Low	Mid	Lowest	Low	Mid	Low	1:4
Low	Low	Mid	Lowest	Low	Mid	Mid	1:4
Low	Low	Mid	Lowest	Low	Mid	High	1:4
Low	Low	Mid	Lowest	Low	High	Mid	1:4
Low	Low	Mid	Lowest	Low	High	High	1:4
Low	Low	Mid	Lowest	Mid	n/a	n/a	n/a
Low	Low	Mid	Lowest	Mid	Low	Low	n/a
Low	Low	Mid	Lowest	Mid	Low	Mid	n/a
Low	Low	Mid	Lowest	Mid	Mid	Mid	n/a
Low	Low	Mid	Lowest	Mid	High	Low	n/a
Low	Low	Mid	Lowest	Mid	High	Mid	n/a
Low	Low	Mid	Lowest	Mid	Low	Low	4:1
Low	Low	Mid	Lowest	Mid	Low	Mid	4:1
Low	Low	Mid	Lowest	Mid	Low	High	4:1
Low	Low	Mid	Lowest	Mid	Mid	Mid	4:1
Low	Low	Mid	Lowest	Mid	Mid	High	4:1
Low	Low	Mid	Lowest	Mid	High	Low	4:1
Low	Low	Mid	Lowest	Mid	High	Mid	4:1
Low	Low	Mid	Lowest	Mid	High	High	4:1
Low	Low	Mid	Lowest	Mid	Low	Low	1:1
Low	Low	Mid	Lowest	Mid	Low	High	1:1
Low	Low	Mid	Lowest	Mid	Mid	Mid	1:1
Low	Low	Mid	Lowest	Mid	Mid	High	1:1
Low	Low	Mid	Lowest	Mid	High	Mid	1:1
Low	Low	Mid	Lowest	Mid	High	High	1:1
Low	Low	Mid	Lowest	Mid	Low	Low	1:4
Low	Low	Mid	Lowest	Mid	Mid	Mid	1:4
Low	Low	Mid	Lowest	Mid	Mid	High	1:4
Low	Low	Mid	Lowest	Mid	High	Mid	1:4
Low	Low	Mid	Lowest	Mid	High	High	1:4
Low	Low	Mid	Lowest	High	n/a	n/a	n/a
Low	Low	Mid	Lowest	High	Low	Low	n/a
Low	Low	Mid	Lowest	High	Low	Mid	n/a
Low	Low	Mid	Lowest	High	Mid	Low	n/a
Low	Low	Mid	Lowest	High	Mid	Mid	n/a
Low	Low	Mid	Lowest	High	High	Low	n/a
Low	Low	Mid	Lowest	High	High	High	n/a

μ	Bottleneck Size	K	Infection Duration	Recombination	Ξ	Burst Size	DFE
Low	Low	Mid	Lowest	High	Low	Low	4:1
Low	Low	Mid	Lowest	High	Low	Mid	4:1
Low	Low	Mid	Lowest	High	Low	High	4:1
Low	Low	Mid	Lowest	High	Mid	Mid	4:1
Low	Low	Mid	Lowest	High	High	Mid	4:1
Low	Low	Mid	Lowest	High	High	High	4:1
Low	Low	Mid	Lowest	High	Low	Mid	1:1
Low	Low	Mid	Lowest	High	Low	High	1:1
Low	Low	Mid	Lowest	High	Mid	High	1:1
Low	Low	Mid	Lowest	High	High	Low	1:1
Low	Low	Mid	Lowest	High	High	Mid	1:1
Low	Low	Mid	Lowest	High	High	High	1:1
Low	Low	Mid	Lowest	High	Low	Mid	1:4
Low	Low	Mid	Lowest	High	Mid	Mid	1:4
Low	Low	Mid	Lowest	High	Mid	High	1:4
Low	Low	Mid	Lowest	High	High	Mid	1:4
Low	Low	Mid	Lowest	High	High	High	1:4
Low	Low	High	Lowest	n/a	n/a	n/a	n/a
Low	Low	High	Lowest	Low	Low	Mid	n/a
Low	Low	High	Lowest	Low	Low	High	n/a
Low	Low	High	Lowest	Low	Mid	Low	n/a
Low	Low	High	Lowest	Low	Mid	Mid	n/a
Low	Low	High	Lowest	Low	High	Low	n/a
Low	Low	High	Lowest	Low	High	High	n/a
Low	Low	High	Lowest	Low	Low	Low	4:1
Low	Low	High	Lowest	Low	Mid	Low	4:1
Low	Low	High	Lowest	Low	Mid	High	4:1
Low	Low	High	Lowest	Low	High	Mid	4:1
Low	Low	High	Lowest	Low	High	High	4:1
Low	Low	High	Lowest	Low	Mid	Low	1:1
Low	Low	High	Lowest	Low	Mid	Mid	1:1
Low	Low	High	Lowest	Low	Mid	High	1:1
Low	Low	High	Lowest	Low	High	Mid	1:1
Low	Low	High	Lowest	Low	High	High	1:1
Low	Low	High	Lowest	Low	Mid	High	1:4
Low	Low	High	Lowest	Low	High	High	1:4
Low	Low	High	Lowest	Mid	n/a	n/a	n/a
Low	Low	High	Lowest	Mid	Low	Low	n/a
Low	Low	High	Lowest	Mid	Low	Mid	n/a
Low	Low	High	Lowest	Mid	Low	High	n/a
Low	Low	High	Lowest	Mid	Mid	Low	n/a

μ	Bottleneck Size	K	Infection Duration	Recombination	Ξ	Burst Size	DFE
Low	Low	High	Lowest	Mid	Mid	Mid	n/a
Low	Low	High	Lowest	Mid	Mid	High	n/a
Low	Low	High	Lowest	Mid	High	Mid	n/a
Low	Low	High	Lowest	Mid	High	High	n/a
Low	Low	High	Lowest	Mid	Low	Low	4:1
Low	Low	High	Lowest	Mid	Low	High	4:1
Low	Low	High	Lowest	Mid	Mid	Mid	4:1
Low	Low	High	Lowest	Mid	Mid	High	4:1
Low	Low	High	Lowest	Mid	High	Low	4:1
Low	Low	High	Lowest	Mid	High	Mid	4:1
Low	Low	High	Lowest	Mid	High	High	4:1
Low	Low	High	Lowest	Mid	Low	Low	1:1
Low	Low	High	Lowest	Mid	Low	Mid	1:1
Low	Low	High	Lowest	Mid	Low	High	1:1
Low	Low	High	Lowest	Mid	Mid	Mid	1:1
Low	Low	High	Lowest	Mid	Mid	High	1:1
Low	Low	High	Lowest	Mid	High	Mid	1:1
Low	Low	High	Lowest	Mid	Mid	High	1:4
Low	Low	High	Lowest	Mid	High	Mid	1:4
Low	Low	High	Lowest	High	n/a	n/a	n/a
Low	Low	High	Lowest	High	Low	Mid	n/a
Low	Low	High	Lowest	High	Mid	Mid	n/a
Low	Low	High	Lowest	High	Mid	High	n/a
Low	Low	High	Lowest	High	High	Low	n/a
Low	Low	High	Lowest	High	High	High	n/a
Low	Low	High	Lowest	High	Low	Low	4:1
Low	Low	High	Lowest	High	Low	Mid	4:1
Low	Low	High	Lowest	High	Mid	Mid	4:1
Low	Low	High	Lowest	High	Mid	High	4:1
Low	Low	High	Lowest	High	High	Low	4:1
Low	Low	High	Lowest	High	High	Mid	4:1
Low	Low	High	Lowest	High	High	High	4:1
Low	Low	High	Lowest	High	Low	Mid	1:1
Low	Low	High	Lowest	High	Mid	Mid	1:1
Low	Low	High	Lowest	High	Mid	High	1:1
Low	Low	High	Lowest	High	High	Mid	1:1
Low	Low	High	Lowest	High	High	High	1:1
Mid	Low	Mid	Lowest	n/a	n/a	n/a	n/a
Mid	Low	Mid	Lowest	Low	n/a	n/a	n/a
Mid	Low	Mid	Lowest	Low	Low	Low	n/a
Mid	Low	Mid	Lowest	Low	Mid	Low	n/a

μ	Bottleneck Size	K	Infection Duration	Recombination	Ξ	Burst Size	DFE
Mid	Low	Mid	Lowest	Low	Low	Low	4:1
Mid	Low	Mid	Lowest	Low	Low	Mid	4:1
Mid	Low	Mid	Lowest	Low	Low	High	4:1
Mid	Low	Mid	Lowest	Low	Mid	Low	4:1
Mid	Low	Mid	Lowest	Low	Low	Low	1:1
Mid	Low	Mid	Lowest	Low	Low	Mid	1:1
Mid	Low	Mid	Lowest	Low	Low	High	1:1
Mid	Low	Mid	Lowest	Low	Mid	Low	1:1
Mid	Low	Mid	Lowest	Low	Low	Low	1:4
Mid	Low	Mid	Lowest	Low	Low	Mid	1:4
Mid	Low	Mid	Lowest	Low	Mid	Low	1:4
Mid	Low	Mid	Lowest	Low	High	Low	1:4
Mid	Low	Mid	Lowest	Mid	n/a	n/a	n/a
Mid	Low	Mid	Lowest	Mid	Low	Low	n/a
Mid	Low	Mid	Lowest	Mid	Low	Mid	n/a
Mid	Low	Mid	Lowest	Mid	Mid	Low	n/a
Mid	Low	Mid	Lowest	Mid	Low	Low	4:1
Mid	Low	Mid	Lowest	Mid	Low	Mid	4:1
Mid	Low	Mid	Lowest	Mid	Mid	Low	4:1
Mid	Low	Mid	Lowest	Mid	Low	Low	1:1
Mid	Low	Mid	Lowest	Mid	Low	Mid	1:1
Mid	Low	Mid	Lowest	Mid	Low	High	1:1
Mid	Low	Mid	Lowest	Mid	Mid	Low	1:1
Mid	Low	Mid	Lowest	Mid	Low	Low	1:4
Mid	Low	Mid	Lowest	Mid	Low	Mid	1:4
Mid	Low	Mid	Lowest	Mid	Low	High	1:4
Mid	Low	Mid	Lowest	Mid	Mid	Low	1:4
Mid	Low	Mid	Lowest	Mid	Mid	Mid	1:4
Mid	Low	Mid	Lowest	Mid	High	Low	1:4
Mid	Low	Mid	Lowest	High	n/a	n/a	n/a
Mid	Low	Mid	Lowest	High	Low	Low	n/a
Mid	Low	Mid	Lowest	High	Low	Mid	n/a
Mid	Low	Mid	Lowest	High	Mid	Low	n/a
Mid	Low	Mid	Lowest	High	Low	Low	4:1
Mid	Low	Mid	Lowest	High	Low	Mid	4:1
Mid	Low	Mid	Lowest	High	Low	High	4:1
Mid	Low	Mid	Lowest	High	Mid	Low	4:1
Mid	Low	Mid	Lowest	High	Low	Low	1:1
Mid	Low	Mid	Lowest	High	Low	Mid	1:1
Mid	Low	Mid	Lowest	High	Mid	Low	1:1
Mid	Low	Mid	Lowest	High	Low	Low	1:4

μ	Bottleneck Size	K	Infection Duration	Recombination	Ξ	Burst Size	DFE
Mid	Low	Mid	Lowest	High	Low	Mid	1:4
Mid	Low	Mid	Lowest	High	Low	High	1:4
Mid	Low	Mid	Lowest	High	Mid	Low	1:4
Mid	Low	Mid	Lowest	High	High	Low	1:4
Mid	Low	High	Lowest	n/a	n/a	n/a	n/a
Mid	Low	High	Lowest	Low	n/a	n/a	n/a
Mid	Low	High	Lowest	Low	Low	Low	n/a
Mid	Low	High	Lowest	Low	Low	Mid	n/a
Mid	Low	High	Lowest	Low	Low	High	n/a
Mid	Low	High	Lowest	Low	Mid	Low	n/a
Mid	Low	High	Lowest	Low	Low	Low	4:1
Mid	Low	High	Lowest	Low	Low	Mid	4:1
Mid	Low	High	Lowest	Low	Low	High	4:1
Mid	Low	High	Lowest	Low	Mid	Low	4:1
Mid	Low	High	Lowest	Low	High	Low	4:1
Mid	Low	High	Lowest	Low	Low	Low	1:1
Mid	Low	High	Lowest	Low	Low	Mid	1:1
Mid	Low	High	Lowest	Low	Low	High	1:1
Mid	Low	High	Lowest	Low	Mid	Low	1:1
Mid	Low	High	Lowest	Low	High	Low	1:1
Mid	Low	High	Lowest	Low	Low	Low	1:4
Mid	Low	High	Lowest	Low	Low	Mid	1:4
Mid	Low	High	Lowest	Low	Low	High	1:4
Mid	Low	High	Lowest	Low	Mid	Low	1:4
Mid	Low	High	Lowest	Low	Mid	Mid	1:4
Mid	Low	High	Lowest	Low	High	Low	1:4
Mid	Low	High	Lowest	Mid	n/a	n/a	n/a
Mid	Low	High	Lowest	Mid	Low	Low	n/a
Mid	Low	High	Lowest	Mid	Low	Mid	n/a
Mid	Low	High	Lowest	Mid	Mid	Low	n/a
Mid	Low	High	Lowest	Mid	Low	Low	4:1
Mid	Low	High	Lowest	Mid	Low	Mid	4:1
Mid	Low	High	Lowest	Mid	Low	High	4:1
Mid	Low	High	Lowest	Mid	Mid	Low	4:1
Mid	Low	High	Lowest	Mid	Low	Low	1:1
Mid	Low	High	Lowest	Mid	Low	Mid	1:1
Mid	Low	High	Lowest	Mid	Low	High	1:1
Mid	Low	High	Lowest	Mid	Mid	Low	1:1
Mid	Low	High	Lowest	Mid	Low	Mid	1:4
Mid	Low	High	Lowest	Mid	Low	High	1:4
Mid	Low	High	Lowest	Mid	Mid	Low	1:4

μ	Bottleneck Size	K	Infection Duration	Recombination	Ξ	Burst Size	DFE
Mid	Low	High	Lowest	Mid	Mid	Mid	1:4
Mid	Low	High	Lowest	Mid	High	Mid	1:4
Mid	Low	High	Lowest	High	n/a	n/a	n/a
Mid	Low	High	Lowest	High	Low	Low	n/a
Mid	Low	High	Lowest	High	Low	Mid	n/a
Mid	Low	High	Lowest	High	Low	High	n/a
Mid	Low	High	Lowest	High	Mid	Low	n/a
Mid	Low	High	Lowest	High	Low	Low	4:1
Mid	Low	High	Lowest	High	Low	Mid	4:1
Mid	Low	High	Lowest	High	Low	High	4:1
Mid	Low	High	Lowest	High	Mid	Low	4:1
Mid	Low	High	Lowest	High	Low	Low	1:1
Mid	Low	High	Lowest	High	Low	Mid	1:1
Mid	Low	High	Lowest	High	Low	High	1:1
Mid	Low	High	Lowest	High	Mid	Low	1:1
Mid	Low	High	Lowest	High	High	Low	1:1
Mid	Low	High	Lowest	High	Low	Low	1:4
Mid	Low	High	Lowest	High	Low	Mid	1:4
Mid	Low	High	Lowest	High	Low	High	1:4
Mid	Low	High	Lowest	High	Mid	Mid	1:4
Mid	Low	High	Lowest	High	High	Low	1:4
High	Low	High	Lowest	Low	Low	Low	1:4
High	Low	High	Lowest	Mid	Low	Low	1:4
High	Low	High	Lowest	High	Low	Mid	1:4
Low	Low	Low	Low	Low	Mid	High	1:1
Low	Low	Low	Low	Low	Low	Low	1:4
Low	Low	Low	Low	Low	Low	Mid	1:4
Low	Low	Low	Low	Low	Low	High	1:4
Low	Low	Low	Low	Low	Mid	Low	1:4
Low	Low	Low	Low	Low	Mid	Mid	1:4
Low	Low	Low	Low	Low	Mid	High	1:4
Low	Low	Low	Low	Low	High	Low	1:4
Low	Low	Low	Low	Low	High	Mid	1:4
Low	Low	Low	Low	Low	High	High	1:4
Low	Low	Low	Low	Mid	Mid	High	1:1
Low	Low	Low	Low	Mid	Low	Low	1:4
Low	Low	Low	Low	Mid	Low	Mid	1:4
Low	Low	Low	Low	Mid	Low	High	1:4
Low	Low	Low	Low	Mid	Mid	Low	1:4
Low	Low	Low	Low	Mid	Mid	Mid	1:4
Low	Low	Low	Low	Mid	Mid	High	1:4

μ	Bottleneck Size	K	Infection Duration	Recombination	Ξ	Burst Size	DFE
Low	Low	Low	Low	Mid	High	Low	1:4
Low	Low	Low	Low	Mid	High	Mid	1:4
Low	Low	Low	Low	Mid	High	High	1:4
Low	Low	Low	Low	High	Mid	High	1:1
Low	Low	Low	Low	High	Low	Low	1:4
Low	Low	Low	Low	High	Low	Mid	1:4
Low	Low	Low	Low	High	Low	High	1:4
Low	Low	Low	Low	High	Mid	Low	1:4
Low	Low	Low	Low	High	Mid	High	1:4
Low	Low	Low	Low	High	High	Low	1:4
Low	Low	Low	Low	High	High	High	1:4
Low	Low	Mid	Low	n/a	n/a	n/a	n/a
Low	Low	Mid	Low	Low	n/a	n/a	n/a
Low	Low	Mid	Low	Low	Low	Low	n/a
Low	Low	Mid	Low	Low	Low	Mid	n/a
Low	Low	Mid	Low	Low	Low	High	n/a
Low	Low	Mid	Low	Low	Mid	Low	n/a
Low	Low	Mid	Low	Low	Low	Mid	4:1
Low	Low	Mid	Low	Low	Low	High	4:1
Low	Low	Mid	Low	Low	Mid	Low	4:1
Low	Low	Mid	Low	Low	Low	Mid	1:1
Low	Low	Mid	Low	Low	Low	High	1:1
Low	Low	Mid	Low	Low	Mid	Low	1:1
Low	Low	Mid	Low	Low	High	Low	1:1
Low	Low	Mid	Low	Low	Low	High	1:4
Low	Low	Mid	Low	Low	Mid	Mid	1:4
Low	Low	Mid	Low	Low	Mid	High	1:4
Low	Low	Mid	Low	Low	High	Low	1:4
Low	Low	Mid	Low	Mid	n/a	n/a	n/a
Low	Low	Mid	Low	Mid	Low	Low	n/a
Low	Low	Mid	Low	Mid	Low	Mid	n/a
Low	Low	Mid	Low	Mid	Low	High	n/a
Low	Low	Mid	Low	Mid	Low	Low	4:1
Low	Low	Mid	Low	Mid	Low	Mid	4:1
Low	Low	Mid	Low	Mid	Low	High	4:1
Low	Low	Mid	Low	Mid	Mid	Low	4:1
Low	Low	Mid	Low	Mid	High	Low	1:1
Low	Low	Mid	Low	Mid	Low	High	1:4
Low	Low	Mid	Low	Mid	Mid	Mid	1:4
Low	Low	Mid	Low	Mid	Mid	High	1:4
Low	Low	Mid	Low	Mid	High	Low	1:4

μ	Bottleneck Size	K	Infection Duration	Recombination	Ξ	Burst Size	DFE
Low	Low	Mid	Low	Mid	High	Mid	1:4
Low	Low	Mid	Low	Mid	High	High	1:4
Low	Low	Mid	Low	High	n/a	n/a	n/a
Low	Low	Mid	Low	High	Low	Low	n/a
Low	Low	Mid	Low	High	Low	Mid	n/a
Low	Low	Mid	Low	High	Low	High	n/a
Low	Low	Mid	Low	High	Low	Mid	4:1
Low	Low	Mid	Low	High	Low	High	4:1
Low	Low	Mid	Low	High	Mid	Low	4:1
Low	Low	Mid	Low	High	Low	Low	1:1
Low	Low	Mid	Low	High	Mid	Low	1:1
Low	Low	Mid	Low	High	Mid	Mid	1:1
Low	Low	Mid	Low	High	Mid	Mid	1:4
Low	Low	Mid	Low	High	Mid	High	1:4
Low	Low	Mid	Low	High	High	Low	1:4
Low	Low	Mid	Low	High	High	Mid	1:4
Low	Low	Mid	Low	High	High	High	1:4
Low	Low	High	Low	Low	n/a	n/a	n/a
Low	Low	High	Low	Low	Low	Low	n/a
Low	Low	High	Low	Low	Low	Mid	n/a
Low	Low	High	Low	Low	Mid	Low	n/a
Low	Low	High	Low	Low	High	Low	n/a
Low	Low	High	Low	Low	Low	Low	4:1
Low	Low	High	Low	Low	Mid	Mid	4:1
Low	Low	High	Low	Low	High	Low	4:1
Low	Low	High	Low	Low	Low	High	1:1
Low	Low	High	Low	Low	Mid	Low	1:1
Low	Low	High	Low	Low	Mid	Mid	1:1
Low	Low	High	Low	Low	High	Low	1:1
Low	Low	High	Low	Low	Mid	Mid	1:4
Low	Low	High	Low	Low	Mid	High	1:4
Low	Low	High	Low	Low	High	Low	1:4
Low	Low	High	Low	Low	High	Mid	1:4
Low	Low	High	Low	Low	High	High	1:4
Low	Low	High	Low	Mid	n/a	n/a	n/a
Low	Low	High	Low	Mid	Low	Low	n/a
Low	Low	High	Low	Mid	Low	High	n/a
Low	Low	High	Low	Mid	Mid	Low	n/a
Low	Low	High	Low	Mid	High	Low	n/a
Low	Low	High	Low	Mid	Low	Low	4:1
Low	Low	High	Low	Mid	Low	Mid	4:1

μ	Bottleneck Size	K	Infection Duration	Recombination	Ξ	Burst Size	DFE
Low	Low	High	Low	Mid	Low	High	4:1
Low	Low	High	Low	Mid	Mid	Low	4:1
Low	Low	High	Low	Mid	Mid	Mid	4:1
Low	Low	High	Low	Mid	High	Low	4:1
Low	Low	High	Low	Mid	Low	Mid	1:1
Low	Low	High	Low	Mid	Mid	Low	1:1
Low	Low	High	Low	Mid	Mid	Mid	1:1
Low	Low	High	Low	Mid	High	Low	1:1
Low	Low	High	Low	Mid	High	Mid	1:1
Low	Low	High	Low	Mid	Low	Low	1:4
Low	Low	High	Low	Mid	Mid	Mid	1:4
Low	Low	High	Low	Mid	Mid	High	1:4
Low	Low	High	Low	Mid	High	Mid	1:4
Low	Low	High	Low	Mid	High	High	1:4
Low	Low	High	Low	High	n/a	n/a	n/a
Low	Low	High	Low	High	Low	Low	n/a
Low	Low	High	Low	High	Low	Mid	n/a
Low	Low	High	Low	High	Mid	Low	n/a
Low	Low	High	Low	High	High	Low	n/a
Low	Low	High	Low	High	Low	Mid	4:1
Low	Low	High	Low	High	Mid	Mid	4:1
Low	Low	High	Low	High	High	Low	4:1
Low	Low	High	Low	High	Low	Low	1:1
Low	Low	High	Low	High	Low	Mid	1:1
Low	Low	High	Low	High	Mid	Mid	1:1
Low	Low	High	Low	High	High	High	1:1
Low	Low	High	Low	High	Low	High	1:4
Low	Low	High	Low	High	Mid	Mid	1:4
Low	Low	High	Low	High	Mid	High	1:4
Low	Low	High	Low	High	High	Mid	1:4
Low	Low	High	Low	High	High	High	1:4
Mid	Low	Mid	Low	Low	Low	Low	1:4
Mid	Low	Mid	Low	Low	Low	Mid	1:4
Mid	Low	Mid	Low	Low	Mid	Low	1:4
Mid	Low	Mid	Low	Mid	Low	Low	1:4
Mid	Low	Mid	Low	Mid	Low	Mid	1:4
Mid	Low	Mid	Low	Mid	Mid	Low	1:4
Mid	Low	Mid	Low	High	Low	Low	1:4
Mid	Low	Mid	Low	High	Low	Mid	1:4
Mid	Low	Mid	Low	High	Mid	Low	1:4
Mid	Low	High	Low	Low	n/a	n/a	n/a

μ	Bottleneck Size	K	Infection Duration	Recombination	Ξ	Burst Size	DFE
Mid	Low	High	Low	Low	Low	Low	n/a
Mid	Low	High	Low	Low	Low	Mid	n/a
Mid	Low	High	Low	Low	Low	Low	4:1
Mid	Low	High	Low	Low	Low	Low	1:1
Mid	Low	High	Low	Low	Low	Low	1:4
Mid	Low	High	Low	Mid	n/a	n/a	n/a
Mid	Low	High	Low	Mid	Low	Low	n/a
Mid	Low	High	Low	Mid	Low	Low	4:1
Mid	Low	High	Low	Mid	Low	Mid	4:1
Mid	Low	High	Low	Mid	Low	Low	1:1
Mid	Low	High	Low	Mid	Low	Mid	1:1
Mid	Low	High	Low	Mid	Mid	Low	1:1
Mid	Low	High	Low	Mid	Low	Low	1:4
Mid	Low	High	Low	Mid	Low	Mid	1:4
Mid	Low	High	Low	Mid	Low	High	1:4
Mid	Low	High	Low	Mid	Mid	Low	1:4
Mid	Low	High	Low	High	n/a	n/a	n/a
Mid	Low	High	Low	High	Low	Low	4:1
Mid	Low	High	Low	High	Low	Low	1:1
Mid	Low	High	Low	High	Low	Mid	1:1
Mid	Low	High	Low	High	Mid	Low	1:1
Mid	Low	High	Low	High	Low	Low	1:4
Mid	Low	High	Low	High	Low	Mid	1:4
Mid	Low	High	Low	High	Low	High	1:4
Mid	Low	High	Low	High	Mid	Low	1:4
Mid	Low	High	Low	High	High	Low	1:4
Low	Low	Low	Mid	Low	Mid	High	1:1
Low	Low	Low	Mid	Low	Mid	Mid	1:4
Low	Low	Low	Mid	Low	Mid	High	1:4
Low	Low	Low	Mid	Mid	Mid	High	1:1
Low	Low	Low	Mid	Mid	Mid	High	1:4
Low	Low	Low	Mid	High	Mid	High	1:1
Low	Low	Low	Mid	High	Low	High	1:4
Low	Low	Low	Mid	High	Mid	High	1:4
Low	Low	Low	Mid	High	High	Mid	1:4
Low	Low	Mid	Mid	n/a	n/a	n/a	n/a
Low	Low	Mid	Mid	Low	n/a	n/a	n/a
Low	Low	Mid	Mid	Low	Low	Low	n/a
Low	Low	Mid	Mid	Low	Low	Low	4:1
Low	Low	Mid	Mid	Low	Mid	Low	4:1
Low	Low	Mid	Mid	Low	Low	Low	1:1

μ	Bottleneck Size	K	Infection Duration	Recombination	Ξ	Burst Size	DFE
Low	Low	Mid	Mid	Low	Low	Mid	1:1
Low	Low	Mid	Mid	Low	Low	High	1:1
Low	Low	Mid	Mid	Low	Mid	Low	1:1
Low	Low	Mid	Mid	Low	Low	Low	1:4
Low	Low	Mid	Mid	Low	Low	Mid	1:4
Low	Low	Mid	Mid	Low	Low	High	1:4
Low	Low	Mid	Mid	Low	Mid	Low	1:4
Low	Low	Mid	Mid	Mid	Low	Low	4:1
Low	Low	Mid	Mid	Mid	Mid	Low	4:1
Low	Low	Mid	Mid	Mid	Low	Low	1:1
Low	Low	Mid	Mid	Mid	Low	Mid	1:1
Low	Low	Mid	Mid	Mid	Mid	Low	1:1
Low	Low	Mid	Mid	Mid	Low	Mid	1:4
Low	Low	Mid	Mid	Mid	Low	High	1:4
Low	Low	Mid	Mid	Mid	Mid	Low	1:4
Low	Low	Mid	Mid	High	n/a	n/a	n/a
Low	Low	Mid	Mid	High	Low	Mid	1:1
Low	Low	Mid	Mid	High	Low	Low	1:4
Low	Low	Mid	Mid	High	Low	Mid	1:4
Low	Low	Mid	Mid	High	Low	High	1:4
Low	Low	Mid	Mid	High	Mid	Low	1:4
Low	Low	High	Mid	n/a	n/a	n/a	n/a
Low	Low	High	Mid	Low	n/a	n/a	n/a
Low	Low	High	Mid	Low	Low	Mid	n/a
Low	Low	High	Mid	Low	Mid	Low	n/a
Low	Low	High	Mid	Low	Low	Mid	4:1
Low	Low	High	Mid	Low	Low	High	4:1
Low	Low	High	Mid	Low	Low	Low	1:1
Low	Low	High	Mid	Low	Low	Mid	1:1
Low	Low	High	Mid	Low	Low	High	1:1
Low	Low	High	Mid	Low	Mid	Low	1:1
Low	Low	High	Mid	Low	Low	Mid	1:4
Low	Low	High	Mid	Low	Mid	Mid	1:4
Low	Low	High	Mid	Low	High	Low	1:4
Low	Low	High	Mid	Mid	n/a	n/a	n/a
Low	Low	High	Mid	Mid	Low	Low	n/a
Low	Low	High	Mid	Mid	Low	Mid	n/a
Low	Low	High	Mid	Mid	Mid	Low	n/a
Low	Low	High	Mid	Mid	Low	Low	4:1
Low	Low	High	Mid	Mid	Low	Low	1:1
Low	Low	High	Mid	Mid	Low	Mid	1:1

μ	Bottleneck Size	K	Infection Duration	Recombination	Ξ	Burst Size	DFE
Low	Low	High	Mid	Mid	Low	High	1:4
Low	Low	High	Mid	Mid	Mid	Mid	1:4
Low	Low	High	Mid	Mid	High	Low	1:4
Low	Low	High	Mid	High	n/a	n/a	n/a
Low	Low	High	Mid	High	Low	Low	n/a
Low	Low	High	Mid	High	Low	Mid	n/a
Low	Low	High	Mid	High	Low	High	n/a
Low	Low	High	Mid	High	Mid	Low	n/a
Low	Low	High	Mid	High	Low	Mid	4:1
Low	Low	High	Mid	High	Low	High	4:1
Low	Low	High	Mid	High	Mid	Low	4:1
Low	Low	High	Mid	High	Low	Low	1:1
Low	Low	High	Mid	High	Low	High	1:1
Low	Low	High	Mid	High	High	Low	1:1
Low	Low	High	Mid	High	Low	High	1:4
Low	Low	High	Mid	High	Mid	Low	1:4
Low	Low	High	Mid	High	Mid	Mid	1:4
Low	Low	High	Mid	High	High	Low	1:4
Mid	Low	High	Mid	Low	Low	Mid	1:4
Mid	Low	High	Mid	Mid	Low	Mid	1:4
Mid	Low	High	Mid	High	Low	Mid	1:4
Low	Low	Low	High	Low	Mid	Mid	1:4
Low	Low	Low	High	Low	Mid	High	1:4
Low	Low	Low	High	Mid	Mid	Mid	1:4
Low	Low	Low	High	Mid	Mid	High	1:4
Low	Low	Low	High	High	Mid	High	1:4
Low	Low	Mid	High	Low	Low	Low	1:4
Low	Low	Mid	High	Low	Low	Mid	1:4
Low	Low	Mid	High	Low	Mid	Low	1:4
Low	Low	Mid	High	Mid	Low	Low	1:4
Low	Low	Mid	High	Mid	Low	Mid	1:4
Low	Low	Mid	High	Mid	Low	High	1:4
Low	Low	Mid	High	Mid	Mid	Low	1:4
Low	Low	Mid	High	High	Low	Low	1:4
Low	Low	Mid	High	High	Low	Mid	1:4
Low	Low	Mid	High	High	Mid	Low	1:4
Low	Low	High	High	n/a	n/a	n/a	n/a
Low	Low	High	High	Low	Low	Low	4:1
Low	Low	High	High	Low	Low	Mid	4:1
Low	Low	High	High	Low	Low	Low	1:1
Low	Low	High	High	Low	Low	Mid	1:1

μ	Bottleneck Size	K	Infection Duration	Recombination	Ξ	Burst Size	DFE
Low	Low	High	High	Low	Mid	Low	1:1
Low	Low	High	High	Low	Low	Mid	1:4
Low	Low	High	High	Low	Low	High	1:4
Low	Low	High	High	Low	Mid	Low	1:4
Low	Low	High	High	Mid	Low	Low	4:1
Low	Low	High	High	Mid	Low	Low	1:1
Low	Low	High	High	Mid	Low	Mid	1:1
Low	Low	High	High	Mid	Mid	Low	1:1
Low	Low	High	High	Mid	Low	Low	1:4
Low	Low	High	High	Mid	Low	Mid	1:4
Low	Low	High	High	Mid	Low	High	1:4
Low	Low	High	High	Mid	Mid	Low	1:4
Low	Low	High	High	High	n/a	n/a	n/a
Low	Low	High	High	High	Low	Low	4:1
Low	Low	High	High	High	Low	Mid	1:1
Low	Low	High	High	High	Mid	Low	1:1
Low	Low	High	High	High	Low	Low	1:4
Low	Low	High	High	High	Low	Mid	1:4
Low	Low	High	High	High	Low	High	1:4
Low	Low	High	High	High	Mid	Low	1:4

Supplemental Table 2. List of models with replicates that initially failed to complete due to exceeding allocated memory.

Mutation Rate (μ)	Carrying Capacity (K)	Bottleneck Size	Infection Duration	Recombination Rate (R)	Progeny Skew Probability (Ξ)	Burst Size	DFE	Missing Reps
High	High	Mid	High	Mid	High	High	n/a	1
High	High	Mid	Mid	High	High	High	n/a	4
High	High	High	Mid	High	High	High	n/a	5
High	High	Mid	High	High	High	High	n/a	2
High	High	High	High	High	High	High	n/a	5
High	High	High	Mid	High	High	High	4,1	1
High	High	Mid	High	High	High	High	4,1	5
High	High	High	High	High	High	High	4,1	3

Supplemental Table 3. Order of models presented in Figure 3, Supplemental Figure 1, and Supplemental Figure 2 as lines (range) and points (mean value). Note that for each figure the mutation rate (μ), initial bottleneck size, and carrying capacity (K) are the same for all models. The infection duration varies between models as described in figure captions.

Order	R	Ξ	Burst Size	DFE
1	n/a	n/a	n/a	n/a
2	Low	n/a	n/a	n/a
3	Mid	n/a	n/a	n/a
4	High	n/a	n/a	n/a
5	Low	Low	Low	n/a
6	Mid	Low	Low	n/a
7	High	Low	Low	n/a
8	Low	Mid	Low	n/a
9	Mid	Mid	Low	n/a
10	High	Mid	Low	n/a
11	Low	High	Low	n/a
12	Mid	High	Low	n/a
13	High	High	Low	n/a
14	Low	Low	Mid	n/a
15	Mid	Low	Mid	n/a
16	High	Low	Mid	n/a
17	Low	Mid	Mid	n/a
18	Mid	Mid	Mid	n/a
19	High	Mid	Mid	n/a
20	Low	High	Mid	n/a
21	Mid	High	Mid	n/a
22	High	High	Mid	n/a
23	Low	Low	High	n/a
24	Mid	Low	High	n/a
25	High	Low	High	n/a
26	Low	Mid	High	n/a
27	Mid	Mid	High	n/a
28	High	Mid	High	n/a
29	Low	High	High	n/a
30	Mid	High	High	n/a
31	High	High	High	n/a
32	Low	Low	Low	4:1
33	Mid	Low	Low	4:1

Order	*R*	*Ξ*	Burst Size	DFE
34	High	Low	Low	4:1
35	Low	Mid	Low	4:1
36	Mid	Mid	Low	4:1
37	High	Mid	Low	4:1
38	Low	High	Low	4:1
39	Mid	High	Low	4:1
40	High	High	Low	4:1
41	Low	Low	Mid	4:1
42	Mid	Low	Mid	4:1
43	High	Low	Mid	4:1
44	Low	Mid	Mid	4:1
45	Mid	Mid	Mid	4:1
46	High	Mid	Mid	4:1
47	Low	High	Mid	4:1
48	Mid	High	Mid	4:1
49	High	High	Mid	4:1
50	Low	Low	High	4:1
51	Mid	Low	High	4:1
52	High	Low	High	4:1
53	Low	Mid	High	4:1
54	Mid	Mid	High	4:1
55	High	Mid	High	4:1
56	Low	High	High	4:1
57	Mid	High	High	4:1
58	High	High	High	4:1
59	Low	Low	Low	1:1
60	Mid	Low	Low	1:1
61	High	Low	Low	1:1
62	Low	Mid	Low	1:1
63	Mid	Mid	Low	1:1
64	High	Mid	Low	1:1
65	Low	High	Low	1:1
66	Mid	High	Low	1:1
67	High	High	Low	1:1
68	Low	Low	Mid	1:1
69	Mid	Low	Mid	1:1
70	High	Low	Mid	1:1
71	Low	Mid	Mid	1:1
72	Mid	Mid	Mid	1:1
73	High	Mid	Mid	1:1
74	Low	High	Mid	1:1

Order	*R*	*Ξ*	Burst Size	DFE
75	Mid	High	Mid	1:1
76	High	High	Mid	1:1
77	Low	Low	High	1:1
78	Mid	Low	High	1:1
79	High	Low	High	1:1
80	Low	Mid	High	1:1
81	Mid	Mid	High	1:1
82	High	Mid	High	1:1
83	Low	High	High	1:1
84	Mid	High	High	1:1
85	High	High	High	1:1
86	Low	Low	Low	1:4
87	Mid	Low	Low	1:4
88	High	Low	Low	1:4
89	Low	Mid	Low	1:4
90	Mid	Mid	Low	1:4
91	High	Mid	Low	1:4
92	Low	High	Low	1:4
93	Mid	High	Low	1:4
94	High	High	Low	1:4
95	Low	Low	Mid	1:4
96	Mid	Low	Mid	1:4
97	High	Low	Mid	1:4
98	Low	Mid	Mid	1:4
99	Mid	Mid	Mid	1:4
100	High	Mid	Mid	1:4
101	Low	High	Mid	1:4
102	Mid	High	Mid	1:4
103	High	High	Mid	1:4
104	Low	Low	High	1:4
105	Mid	Low	High	1:4
106	High	Low	High	1:4
107	Low	Mid	High	1:4
108	Mid	Mid	High	1:4
109	High	Mid	High	1:4
110	Low	High	High	1:4
111	Mid	High	High	1:4
112	High	High	High	1:4

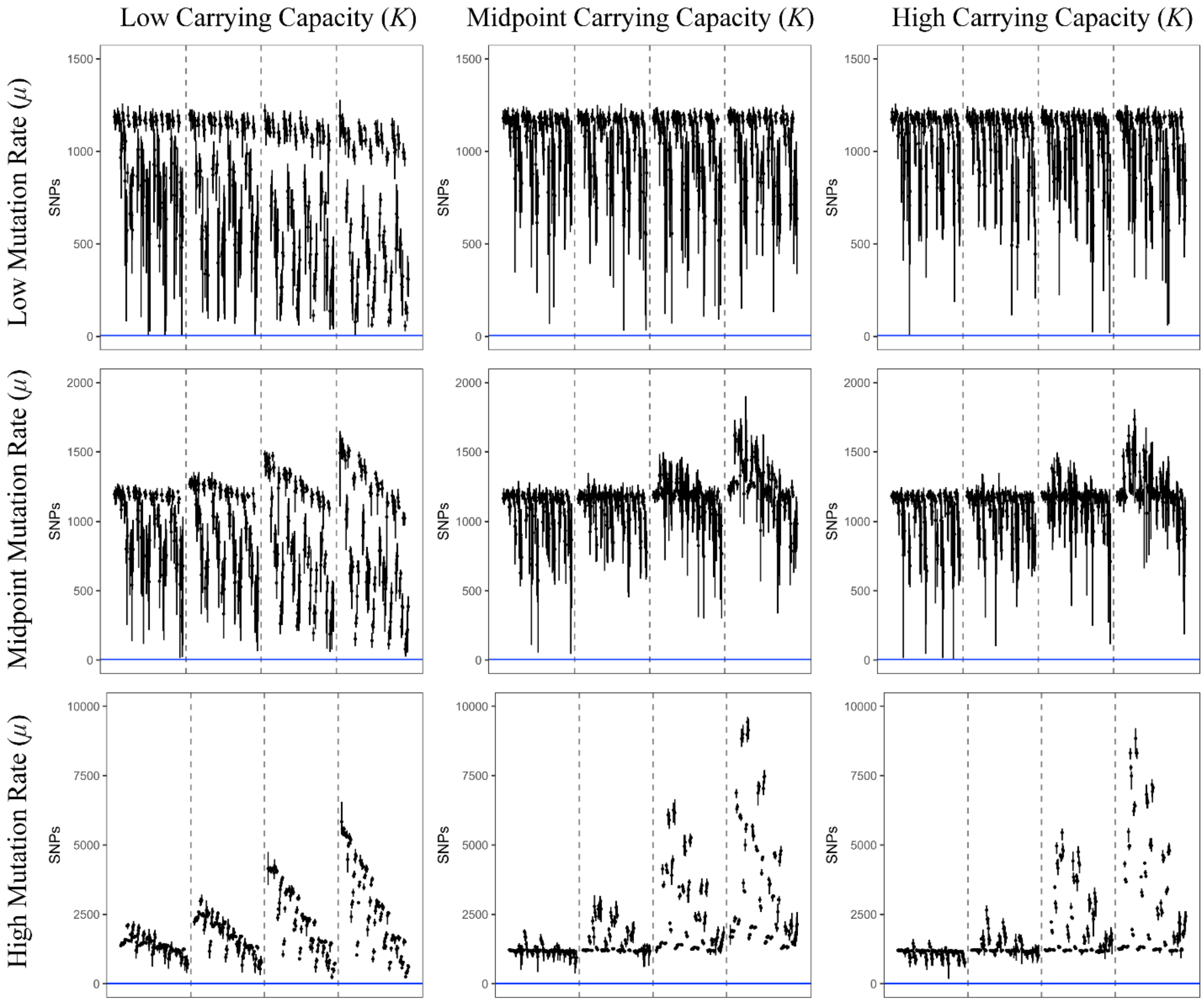

Supplemental Figure 1. Each line represents the range of filtered SNPs for a particular model using a sampling of 1000 genomes; each point is the mean of that model's replicates. All models in this figure used the midpoint bottleneck size (*i.e.*, 5); panels increase from left to right in the value of carrying capacity used and increase from top to bottom in the value of mutation rate used. Within each panel, dashed lines separate models into subpanels with different infection durations, increasing from left to right. Within each subpanel the order of the models is the same and is detailed in Supplemental Table 3. The blue, horizontal line represents the threshold of 5 SNPs used in this study to accept or reject a potential model (note that Y-axes differ by row).

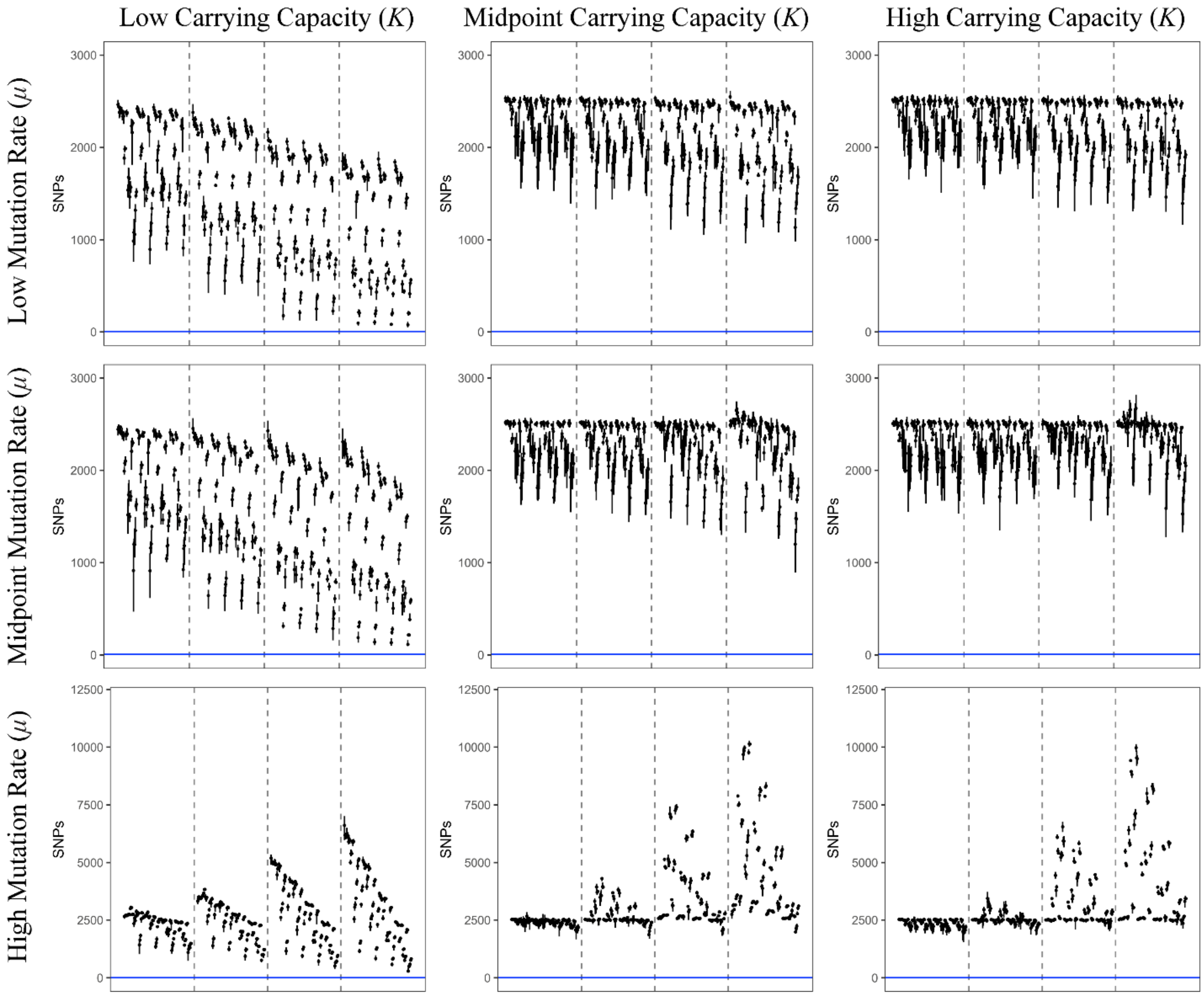

Supplemental Figure 2. Each line represents the range of filtered SNPs for a particular model using a sampling of 1000 genomes; each point is the mean of that model's replicates. All models in this figure used the high bottleneck size (*i.e.*, 100); panels increase from left to right in the value of carrying capacity used and increase from top to bottom in the value of mutation rate used. Within each panel, dashed lines separate models into subpanels with different infection durations, increasing from left to right. Within each subpanel the order of the models is the same and is detailed in Supplemental Table 3. The blue, horizontal line represents the threshold of 5 SNPs used in this study to accept or reject a potential model (note that Y-axes differ by row).
